# Toward Thermally Stimuli-Responsive Polymeric Vesicles Fabricated by Block Copolymer Blends for Nanocarriers

**DOI:** 10.3390/mi16101131

**Published:** 2025-09-30

**Authors:** Jun-Ki Lee, Seung-Bum Heo, Jong Dae Jang, Dong Chul Yang, Dae-Hee Yoon, Changwoo Do, Tae-Hwan Kim

**Affiliations:** 1 Department of Applied Plasma & Quantum Beam Engineering, Jeonbuk National University, Jeonju 54896, Jeonbuk State, Republic of Korea; junki.lee@jbnu.ac.kr (J.-K.L.); yangdong96@jbnu.ac.kr (D.C.Y.); 2 QuantumCat Co., Ltd., 49, Techno-8ro, Yuseong-gu, Daejeon 34028, Republic of Korea; 3 Department of Quantum System Engineering, Jeonbuk National University, Jeonju 54896, Jeonbuk State, Republic of Korea; heobeom0802@jbnu.ac.kr; 4 Neutron Science Division, Korea Atomic Energy Research Institute, 111, Daedeok-daero 989 Beon-gil, Yuseong-gu, Daejeon 34057, Republic of Korea; jdjang@kaeri.re.kr; 5 Division of Electronics and Information Engineering, Jeonbuk National University, Jeonju 54896, Jeonbuk State, Republic of Korea; daehee@jbnu.ac.kr; 6 Neutron Scattering Division, Neutron Sciences Directorate, Oak Ridge National Laboratory, Oak Ridge, TN 37831, USA; doc1@ornl.gov; 7 Department of JBNU-KIST Industry-Academia Convergence Research, Jeonbuk National University, Jeonju 54896, Jeonbuk State, Republic of Korea; 8 Research Center for Advanced Nuclear Interdisciplinary Technology, Jeonbuk National University, Jeonju 54896, Jeonbuk State, Republic of Korea; 9 High-Enthalphy Plasma Research Center, Jeonbuk National University, 546, Bongdong-ro, Bongdong-eup, Wanju-gun 55317, Jeonbuk State, Republic of Korea

**Keywords:** self-assembly, block copolymers, polymer vesicles, phase behavior, small-angle neutron scattering (SANS)

## Abstract

Polymeric vesicles, characterized by enhanced colloidal stability, excellent mechanical properties, controllable surface functionality, and adjustable membrane thickness, are extremely useful in nano- and bio-technology for potential applications as nanosized carriers for drugs and enzymes. However, a few preparative steps are necessary to achieve a unilamellar vesicle with a narrow size distribution. Herein, we report the spontaneous formation of unilamellar polymeric vesicles with nanometer sizes (<50 nm), fabricated by simply mixing diblock copolymers (P(EO-AGE)(2K-2K) and P(EO-AGE)(0.75K-2K)) with differing hydrophilic mass fractions in aqueous solutions. Depending on the mixing ratio of block copolymers and the temperature, the block copolymer mixtures self-assemble into various nanostructures, such as spherical and cylindrical micelles, or vesicles. The self-assembled structures of the block copolymer mixtures were characterized by small-angle neutron scattering, resulting in a phase diagram drawn as a function of temperature and the mixing condition. Notably, the critical temperature for the micelle-to-vesicle phase transition can be easily controlled by altering the mixing conditions; it decreases with an increase in the concentration of one of the block copolymers.

## 1. Introduction

Amphiphilic block copolymers in aqueous solution can self-assemble into various structures, such as spherical or cylindrical micelles, lamellae, and vesicles above the critical micelle concentration, leading to rich phase behavior [1,2,3]. Moreover, the properties of the block copolymers with various self-assembled structures can be controlled or enhanced through polymer synthetic engineering [4,5]. Consequently, amphiphilic block copolymers hold many potential applications, such as nanoscale building blocks or drug carriers in nano-, bio-technology, or pharmaceutical fields [6,7,8,9,10]. Particularly, a polymer vesicular structure can be used for drug carriers with a thermo-responsive release property of the encapsulated therapeutics [11,12,13], cosmetics for skin aesthetics [14,15], nanoreactors with different reaction temperatures [16,17], and diagnostics [18,19], due to the enhanced colloidal stability and mechanical properties, controllable surface functionality, and adjustable membrane thickness. Additionally, these polymeric vesicles exhibit higher stability than the liposomal formulations (Doxil^®^, AmBisome^®^, etc.), making them easier to store. Furthermore, when synthesized with a unilamellar vesicle structure, they offer the advantage of eliminating the need for methods such as extrusion.

The self-assembly of amphiphilic block copolymers into micellar structures such as spherical micelles, cylindrical micelles, or vesicles is primarily driven by the segregation of insoluble and soluble blocks in selective solvents, where the resulting morphology is critically determined by the balance between core surface energy and corona chain stretching, which in turn is highly sensitive to the relative block lengths, especially the volume fraction and conformational entropy of the hydrophilic block. As a result, among various factors, the hydrophilic mass fraction of the block copolymer (f_hydrophilic_) plays a key role in determining the resulting morphology [20,21,22,23]. For example, amphiphilic block copolymers form polymeric vesicles when 0.25 < f_hydrophilic_ < 0.5 and a micellar structure when f_hydrophilic_ > 0.5. However, obtaining unilamellar vesicles with narrow size distributions proves difficult compared to micelles or multilamellar vesicles, despite their importance for studying membrane properties and interactions with biomolecules or colloids. Although some studies have introduced a novel method for unilamellar vesicle fabrication, achieving unilamellar vesicles with a narrow size distribution still demands meticulous preparation techniques (e.g., electroforming, extrusion, heat treatment, or ultrasonic treatment) [24,25,26,27]. Moreover, the common methods for fabricating polymeric vesicles (precipitating block copolymers from solvents or rehydrating a dried film of the block copolymers) generally result in a wide range of size distributions and poor encapsulation performance [28,29,30]. Consequently, crafting unilamellar vesicles with a narrow size distribution remains a significant challenge.

Here, we report a simple method for fabricating spontaneous and temperature-responsive unilamellar polymeric vesicles with a diameter of less than 50 nm by mixing the amphiphilic block copolymers in an aqueous solution, eliminating the need for the complex preparation procedures typical of conventional methods. To prepare these unilamellar vesicles, we mixed block copolymers Poly(ethylene oxide-*b*-allyl glycidyl ether)(2K-2K), P(EO-AGE)(2K-2K) and Poly(ethylene oxide-*b*-allyl glycidyl ether)(0.75K-2K), P(EO-AGE)(0.75K-2K) in aqueous solutions, which have differing hydrophilic mass fractions, leading to a phase transition from micelle to vesicle.

When P(EO-AGE)(2K-2K) is mixed with P(EO-AGE)(0.75K-2K), the f_hydrophilic_ of the polymer micelle is easily decreased without synthesizing a new polymer through chemical reactions. Considering that it is energetically rather challenging to be self-assembled into the vesicles with multiple layers, we expected the formation of unilamellar vesicles to be more natural [31]. Dynamic light scattering (DLS) and small-angle neutron scattering (SANS) confirmed that the mixture of P(EO-AGE)(2K-2K)/P(EO-AGE)(0.75K-2K) self-assembled into either spherical or cylindrical micelle structures, which then transformed into polymeric unilamellar vesicles depending on the temperature and mixing ratio. Additionally, the transition temperature from micelle to vesicle was lowered by increasing the concentration of P(EO-AGE)(0.75K-2K). To the best of our knowledge, this is the first demonstration of spontaneously prepared and small-sized unilamellar vesicles (<50 nm) with a simple procedure using only diblock copolymers in aqueous solutions. Additionally, in previous approaches, forming diverse micellar structures and phase transition control generally required additional steps, such as polymer engineering or adding derivatives. In contrast, this study demonstrates the advantage of achieving micelle structures and phase transition control in a relatively straightforward manner by mixing polymers of identical composition but different chain lengths. Moreover, the simplicity of this approach, coupled with the temperature-responsive self-assembled structures (shape and size) and the biocompatibility of the block copolymers, could enable the production of unilamellar polymeric vesicles with potential applications in drug delivery systems, metabolic engineering, and nanoreactors.

## 2. Materials and Methods

### 2.1. Materials

Poly(ethylene oxide) methyl ether polymer (mPEO; number average molecular weight, Mn = 2000 and 750 g/mol), a monomer of allyl glycidyl ether (AGE; weight average molecular weight, Mw = 114.144 g/mol), potassium cube (in mineral oil), naphthalene (99%), 2.0 M butyl magnesium chloride (in tetrahydrofuran, THF) solution, 1.4 M sec–butyllithium (in cyclohexane) solution and anhydrous hexane (99%) were purchased from Sigma-Aldrich (St. Louis, MO, USA). Tetrahydrofuran (THF) was obtained from Junsei (Tokyo, Japan). Anhydrous methanol (99.9%) was purchased from Alfa Aesar (Ward Hill, MA, USA). D_2_O (99.9 mol% deuterium enriched) was purchased from Cambridge Isotope Laboratory (Tewksbury, MA, USA). H_2_O was purified using a Millipore Direct Q system (Frankfurter, Darmstadt, Germany) immediately before use. All chemicals except THF and AGE were used without further purification.

AGE and THF need a purification process to synthesize the monodispersed block copolymers. For the purification of the synthesized AGE monomer, a 2.0 M solution of butyl magnesium chloride was used for 30 min. It was then degassed by three freeze–pump–thaw cycles under vacuum. To purify THF, it was intensively stirred with a mixture of THF and 1.4 M sec-butyllithium for 30 min and subsequently subjected to three freeze–pump–thaw cycles to remove impurities.

### 2.2. Synthesis of P(EO-AGE) Diblock Copolymers

P(EO-AGE) diblock copolymers were synthesized using living anionic ring-opening polymerization (LAROP) [32]. The LAROP process is highly sensitive to oxygen and humidity; consequently, all experiments were performed under a vacuum. mPEO polymer blocks, weighing 5 g and 1 g (for P(EO-AGE)(2K-2K) and P(EO-AGE)(0.75K-2K), respectively), were purified in a vacuum reactor at 45 °C while being continuously stirred. Subsequently, about 5 mL of a 0.4 M potassium naphthalenide solution (initiator) was injected into the vacuum reactor, followed by the direct addition of 10 mL of purified THF. Upon injection of the initiator, the mPEO solution turned dark green, and the initiation reaction continued for 30 min. Subsequently, AGE monomers weighing 5 g and 20 g (for P(EO-AGE)(2K-2K) and P(EO-AGE)(0.75K-2K), respectively) were then introduced to the mPEO solutions. The reaction of this mixture lasted for 20 h, after which the solution turned light brown. The reaction was halted using anhydrous methyl alcohol. Figure S1 (Supporting Information) illustrates the synthesis scheme of P(EO-AGE) diblock copolymers. The final solutions were precipitated with hexane, and the residual hexane in the resulting block copolymer was evaporated under a vacuum for at least one day. The synthesized P(EO-AGE)(2K-2K) and P(EO-AGE)(0.75K-2K) deblock copolymers were verified by the NMR and GPC measurements (Figures S2 and S3, Supporting Information).

### 2.3. Sample Preparation

The P(E-A)-*x* and P(E-A)-*y*-*z* mixtures were prepared by simply mixing P(EO-AGE)(2K-2K) and P(EO-AGE)(0.75K-2K) in aqueous solution. For the P(E-A)-*x* mixtures, the concentration of P(EO-AGE)(2K-2K) was maintained at 0.1% by weight, with variation in P(EO-AGE)(0.75K-2K) from 0% to 0.2% by weight. For the P(E-A)-*y*-*z* mixtures, the concentration of P(EO-AGE)(2K-2K)/P(EO-AGE)(0.75K-2K) mixtures was fixed at 0.1% or 0.2% by weight, and the ratio of P(EO-AGE)(2K-2K) and P(EO-AGE)(0.75K-2K) was adjusted to 3:1, 2:1, 1:1, 1:2, and 1:3 by mass.

### 2.4. Small-Angle Neutron Scattering (SANS) Measurements

SANS intensities were recorded using the EQ-SANS instrument at the Spallation Neutron Source (SNS) in the United States [33] and the 40 m SANS instrument at the HANARO cold neutron research facility in the Republic of Korea [34]. Neutrons with wavelength band defined by the minimum wavelength (λ) of 2.5 Å and a maximum of 12.0 Å (at 60 Hz operation) in the EQ-SANS experiments, were utilized to examine two different of sample to detector distances (SDDs) of 2.5 m and 4 m, respectively, allowing exploration of the scattered *q* range (0.005 Å^−1^ < *q* < 0.3 Å^−1^), where *q* = (4π/*λ*)sin(*θ*/2) represents the magnitude of the scattering vector, and *θ* is the scattering angle. In the 40 m SANS experiments, neutrons of wavelength (λ) were utilized at λ = 7.49 Å (wavelength spread Δλ/λ = 12%). Three different SDDs (1.16 m, 4.7 m, and 19.8 m) were employed to span the *q* range of 0.001 Å^−1^ < *q* < 0.45 Å^−1^. All the measured sample scattering intensities were adjusted for the background, empty cell scattering, and sensitivity of individual detector pixels. The adjusted sample datasets were calibrated on an absolute scale using the data reduction software provided by HANARO and SNS [35,36] through the standard sample calibration method.

### 2.5. Dynamic Light Scattering (DLS) Measurements

DLS measurements were performed using a ZetaPlus particle size analyzer (λ = 659 nm, scattering angle = 90°, Brookhaven Instruments Corporation, Holtsville, NY, USA).

### 2.6. Transmission Electron Microscopy (TEM) Measurements

TEM images of the P(E-A)-0.2 mixture samples were obtained using high-resolution TEM instruments (Talos F200X G2, Thermo Scientific, Waltham, MA, USA) in the Center for University-wide Research Facilities (CURF) at Jeonbuk National University. A drop of the P(E-A)-0.2 mixture (10 μL) was placed onto a TEM grid using a pipette and then dried at 25 °C and 55 °C.

## 3. Results and Discussion

To facilitate the phase transition from micelle to vesicle, we mixed the block copolymers with varying hydrophilic mass fractions, namely P(EO-AGE)(2K-2K) and P(EO-AGE)(0.75K-2K), where EO and AGE blocks are hydrophilic and hydrophobic blocks, respectively, and their chemical structures are shown in Figure S1 (Supporting Information). In fact, the structure of the block copolymer with an amphiphilicity is determined with the hydrophilic mass fraction of the block copolymer (f_hydrophilic_), which is well-known [23,37]. Consequently, we varied the mixing ratios of these block copolymers to control their effective hydrophilic mass fractions. We hypothesize that the P(EO-AGE)(0.75K-2K) with a relatively low f_hydrophilic_ will more readily induces the phase transition (Herein, we confirmed that the P(EO-AGE)(0.75K-2K) block copolymers alone self-assembled into rather large unilamellar polymeric vesicles (≥100 nm above 40 °C for 0.1~0.2 wt% and the dispersity of 0.29~0.4) (Figures S4 and S5, and Table S1) for all the concentration (0.05~0.2 wt%) and temperature (25~60 °C) ranges we studied in this work. Therefore, the micellar structure (sphere or cylinder) can lead to the self-assembly into different structures when the P(EO-AGE)(2K-2K) (f_hydrophilic_ = 0.5) is mixed with P(EO-AGE)(0.75K-2K), thereby resulting in a phase transition. Moreover, increasing the temperature further enhances the hydrophobic characteristics of the block copolymer mixture, promoting the phase transition that is more likely to occur. To examine the systematic phase behavior in P(EO-AGE)(2K-2K)/P(EO-AGE)(0.75K-2K) mixtures, we prepared two sets of the mixtures: one varying only the concentration of P(EO-AGE)(0.75K-2K) and the other both the concentration and the ratio in P(EO-AGE)(2K-2K)/P(EO-AGE)(0.75K-2K) mixtures. For the single concentration control of P(EO-AGE)(0.75K-2K) (0~0.2 wt%), we maintained the P(EO-AGE)(2K-2K) concentration constant at 0.1 wt% to observe the effect of increasing the hydrophilic component (referred to as the P(E-A)-*x* mixture, where *x* denotes the concentration of P(EO-AGE)(0.75K-2K)). Conversely, in the variable ratio experiment of P(EO-AGE)(2K-2K)/P(EO-AGE)(0.75K-2K) (ratios of 3:1, 2:1, 1:1, 1:2 and 1:3), we fixed the total concentration of the mixtures at 0.1 and 0.2 wt% to examine the implications of altering the hydrophilic component without varying the total concentration (referred to as the P(E-A)-*y*-*z* mixture, where *y* and *z* represent the total concentration and the mixing ratio of P(EO-AGE)(2K-2K)/P(EO-AGE)(0.75K-2K), respectively).

**Figure 1:**
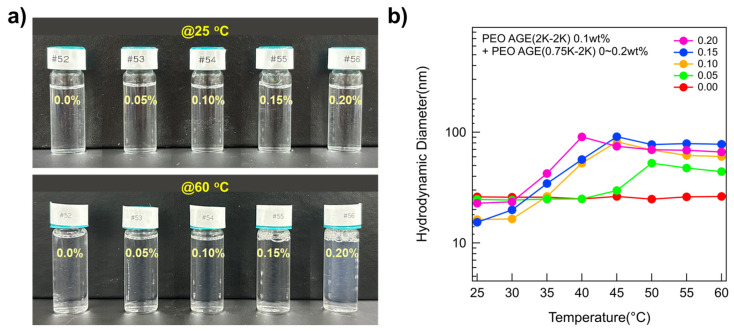
Characteristics of the P(E-A)-*x* mixtures. (**a**) Photos of the P(E-A)-*x* mixtures with *x* = 0~0.2 wt% at 25 °C and 60 °C. (**b**) Hydrodynamic diameter of the P(E-A)-*x* mixtures with *x* = 0~0.2 wt% across temperatures ranging from 25 °C to 60 °C in water.

While all the P(E-A)-*x* mixtures at 25 °C were clear and transparent regardless of *x*, the transparency of the P(E-A)-*x* mixtures at 60 °C varied depending on *x*, appearing somewhat bluish and opaque above 0.1 wt% of *x*. This phenomenon is attributable to the Tyndall effect [38,39,40,41], indicating that the P(E-A)-*x* mixture solutions at 60 °C (*x* ≥ 0.1 wt%) contain large aggregates exceeding 10 nm (Figure 1a). To determine the size of aggregates formed by the P(E-A)-*x* mixture in aqueous solution, dynamic light scattering (DLS) measurements were conducted using as-prepared samples without dilution. The hydrodynamic diameters of P(E-A)-*x* mixtures at 25 °C remained below 25 nm, a typical size for polymeric spherical micelles. Excluding the P(E-A)-*0* mixture, the hydrodynamic diameters of all the P(E-A)-*x* mixtures increased with temperature (44~90 nm) (Figure 1b), consistent with visual observation results. Notably, the hydrodynamic diameter of the P(E-A)-*x* mixtures with relatively high *x* value (above 0.1 wt%) increases with temperature. This indicates that the structure of the P(E-A)-*x* mixtures with relatively high *x* value is highly sensitive to temperature change, i.e., a phase transition occurs more readily at lower temperatures as the concentration of P(EO-AGE)(0.75K-2K) block copolymer increases.

**Figure 2:**
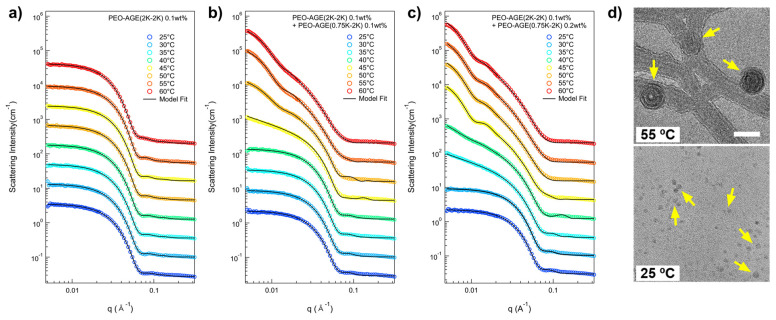
SANS intensities of the P(E-A)-*x* mixtures at various temperatures (25 °C to 60 °C) with (**a**) *x* = 0 wt%, (**b**) *x* = 0.1 wt%, (**c**) *x* = 0.2 wt% and TEM results of P(E-A)-*0.2* mixtures at (**d**) 25 °C and 55 °C. For visual clarity, SANS intensities were vertically shifted. Yellow arrows in (**d**) indicate vesicles and spherical micelles, respectively. The scale bar is 50 and 20 nm for the above and the bottom, respectively.

To elucidate the intricate nanostructure of the P(E-A)-*x* mixture in aqueous solution as influenced by *x* and temperature, a series of small-angle neutron scattering (SANS) measurements was conducted. In the SANS measurement, all the P(E-A)-*x* mixtures were prepared in D_2_O to ensure adequate coherent scattering intensity, visibly demonstrating phase behavior analogous to those mixtures prepared in H_2_O. The SANS intensities for the P(E-A)-*x* mixtures (*x* = 0, 0.05, 0.1, 0.15, and 0.2 wt%) in D_2_O across varying temperatures (25~60 °C) are depicted in Figure 2 and Figure S6. The SANS intensities for the P(E-A)-*0* mixture in D_2_O at different temperatures were nearly identical, exhibiting strong form factor scattering arising from polymer aggregates (characterized by flat (= Q^−0^ behavior) in the low Q region (Q < 0.01 Å^−1^) and Q^−4^ behavior in the middle Q region (0.03 Å^−1^ < Q < 0.07 Å^−1^), typical for a spherical particle [42,43]), indicating no phase transitions within the measured temperature ranges (Figure 2a). Here, Q denotes the magnitude of the scattering vector, defined as Q = (4π/*λ*)sin(*θ*/2), where θ is the scattering angle. Conversely, the SANS intensities for the P(E-A)-*x* mixtures with *x* > 0 did not exhibit a flat response in the low Q region beyond a specific temperature. The P(E-A)-*0.05*, P(E-A)-*0.1*, P(E-A)-*0.15* and P(E-A)-*0.2* mixtures at temperatures of 55~60 °C, 45~55 °C, 40~45 °C and 35~40 °C (Figure 2b,c and Figure S6a,b), displayed the scattering intensities in the low Q region characterized by a Q^−*a*^ behavior (where 0.5 < *a* < 2 and *a* ≠ 1), suggesting the coexistence of nanoparticles of varying shape. As the temperature increased further, the SANS intensity for P(E-A)-*0.1*, P(E-A)-*0.15*, and P(E-A)-*0.2* mixtures exhibited nearly a Q^−2^ behavior in the low Q region above 60 °C, 50 °C, and 45 °C, respectively, indicative of aggregates with a platelike structure such as lamellae or vesicles [44,45]. It is important to note that the transition temperature (Q^−*a*^ behavior, where *a* shifts from 0 to 0.5 < *a* < 2 and finally to 2) is strongly influenced by the concentration of P(EO-AGE)(0.75K-2K) block copolymer (with higher concentration tending to lower transition temperature), which is consistent with the result of DLS measurements. Given that the hydrophobicity of the PEO block intensifies with temperature [46,47], we believe that the effective f_hydrophilic_ attributes of the P(E-A)-*x* mixture can be modulated through a combined effect of temperature and the concentration of additive block copolymers, facilitating its phase transition.

**Figure 3:**
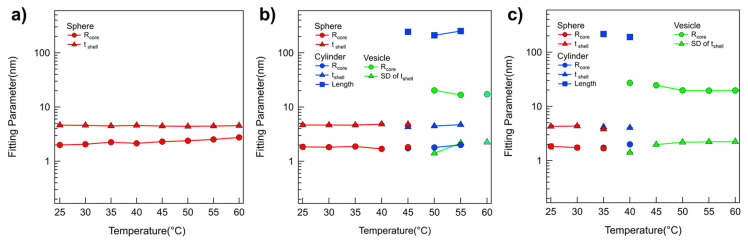
Model fitting results obtained from SANS analysis. Fitting parameters obtained from the form factor fits of SANS intensities of the P(E-A)-*x* mixtures at concentrations of (**a**) *x* = 0 wt%, (**b**) 0.1 wt%, and (**c**) 0.2 wt% in temperature ranges of 25 °C to 60 °C. (Error bars are included in every point, but they may not be visible because they are smaller than the symbol.).

A quantitative analysis of the SANS intensities of P(E-A)-*x* mixtures in D_2_O was conducted using the non-linear least squares model fitting with various numerical functions [48,49,50,51]. All samples were sufficiently diluted to avoid interparticle interaction in the solution; no interparticle interference was detected in any of the SANS intensities. Therefore, only a model fit analysis with an appropriate form factor was considered necessary. In the SANS analysis, the scattering length densities (SLDs) for PEO, PAGE, and D_2_O were used to 0.32 × 10^−6^ Å^−2^, 0.72 × 10^−6^ Å^−2^, and 6.4 × 10^−6^ Å^−2^, respectively, and were basically fixed for the SANS analyses. For the SANS intensities of the P(E-A)-*0* mixture across all temperatures and the P(E-A)-*x* mixtures in D_2_O mixtures at temperatures ranging from 25 °C to 50 °C, which appeared flat in the low Q region, a form factor corresponding to a core–shell spherical particle shape [42,43] was chosen. Considering the solvent affinity of the AGE and the EO blocks, these were designated as the core and shell regions, respectively. The SLD of the core region was set based on the AGE block. In contrast, the SLD of the shell region was adjustable within the range between PEO and D_2_O due to slight water penetration into the hydrophilic EO region. Consequently, the SANS intensities of the P(E-A)-*0* mixture across all temperatures and the P(E-A)-*x* mixtures in D_2_O mixtures at 25 °C to 60 °C were accurately replicated using a core–shell spherical form factor with a core radius R_core_ ranging from 1.87 to 2.9 nm and a shell thickness t_shell_ between 4.1 and 4.9 nm (Figure 2 and Figure 3, Figures S6 and S7). As the temperature increased, the core radius slightly expanded while the shell thickness decreased, reflecting an increase in the hydrophobicity of the EO blocks that led to a reduction in the f_hydrophilc_.

In the SANS intensities of P(E-A)-*x* mixtures at temperature ranges of 55~60 °C, 45~55 °C, 40~45 °C, and 35~40 °C for *x* = 0.05, 0.1, 0.15, and 0.2 wt%, respectively, the form factor model with a singular shape was inconsistent. This discrepancy indicates a mixed phase in the solution. Consequently, a sum model comprising a core–shell spherical and a core–shell cylindrical form factor for P(E-A)-*x* mixtures at 55, 45, 40 and 35 °C for *x* = 0.05, 0.1, 0.15 and 0.2 wt%, respectively, or a core–shell cylindrical form factor combined with a vesicular form factor featuring a shell with Gaussian distribution for P(E-A)-*x* mixtures at 60 °C, 50~55 °C, 45 °C and 40 °C for *x* = 0.05, 0.1, 0.15 and 0.2 wt%, respectively [42,43], was successfully utilized to match the observed SANS intensities (Figure 2b,c and Figure S6a,b). The relevant fitting parameters, including the core radius and the shell thickness for spherical particles, the core radius and shell length for cylindrical particles, and the core radius and the standard deviation of the shell thickness with Gaussian distribution for vesicles, are documented in Figure 3b,c and Figure S7. For P(E-A)-*x* mixtures at 60 °C, 50~60 °C and 45~60 °C for *x* = 0.1, 0.15 and 0.2 wt%, the vesicular form factor with a Gaussian-distributed (where the core radius and the SD of the shell thickness range between 13.2~24.5 nm and 2.0~2.3 nm, respectively) is in complete agreement with the SANS intensities (Figure 2b,c and Figure 3b,c, Figures S6b and S7b). Herein, the dispersity of the radius in the vesicle only ranged from 0.26 to 0.5, indicating a partially narrow size distribution (Table S2). Significantly, while the lamellar form factor failed to correspond with the SANS intensities, this result confirms the formation of a unilamellar vesicle in the mixture. It is important to note that the core radius and the SD of shell thickness of the vesicles showed minor variations with heating, likely due to changes in f_hydrophilic_ values. Still, the overall vesicular structure was preserved without any topological phase transition. Given that the flexible PEO chains with more than 10 monomers in water typically have a monomer length of approximately 0.2 nm [52], the total PEO chain of P(EO-AGE)(2K-2K) containing 46 EO monomers is estimated to measure around 9.2 nm. Considering that the block copolymer in the vesicle exists as a bilayer form, the total PEO chain (ca. 18.4 nm = 9.2 nm × 2) closely matches the full width of the Gaussian distribution (2 × 3 times SD of the shell thickness = 12 nm~13.8 nm). Since the block copolymers forming the bilayer are usually not fully stretched, the bilayer thickness can be slightly smaller than the estimated value. Notably, the temperature dependent phase behavior of P(E-A)-*x* mixtures was reversible. As representative data, we confirmed that the self-assembled structure of P(E-A)-*0.2* mixtures was re-assembled into the same cylindrical and spherical micellar structure after cooling (Figure S6c).

Transmission electron microscopy (TEM) images of P(E-A)-*0.2* mixture at different temperatures (55 °C and 25 °C) further support its phase behavior depending on temperature. The TEM image of P(E-A)-*0.2* mixture at 55 °C clearly showed a platelike shape, which indicates a unilamellar vesicle (Figure 2d above). The overall size of the vesicle obtained from the TEM image was ca. 45 nm, which was comparable to the SANS result (ca. 52 nm, where 52 nm = ((vesicle inner radius (20 nm) + shell thickness (2 nm × 3)) × 2)). For the P(E-A)-*0.2* mixture at 25 °C, the TEM image showed a spherical dot, indicating a spherical micelle (Figure 2d bottom). The size of the micelle obtained from the TEM image was ca. 8 nm, which was also comparable to the SANS result (ca. 12 nm). Both spherical micelles and vesicle sizes obtained from the TEM image are slightly smaller than the SANS results, which can be explained by the difference in the sample preparation for each measurement.

**Figure 4:**
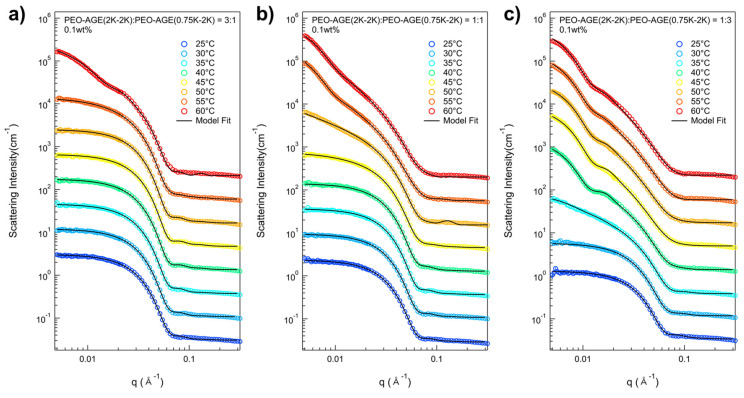
SANS intensities of the P(E-A)-*0.1*-*z* mixtures in D_2_O across a range of (**a**) *z* = 3:1, (**b**) 1:1 and (**c**) 1:3 upon heating (25~60 °C). The SANS intensities were vertically shifted for better visual clarity.

As previously mentioned, the P(E-A)-*y*-*z* mixtures were prepared at 0.1 and 0.2 wt% (where *y* and *z* represent the total concentration and the mixing ratio of P(EO-AGE)(2K-2K)/P(EO-AGE)(0.75K-2K), respectively) in the total concentration to confirm the effect of increasing the hydrophilic moiety without changing the total concentration. The temperature dependent phase behaviors of the P(E-A)-*y*-*z* mixtures were also confirmed through a series of SANS measurements within a temperature range of 25~60 °C. Representative data include the SANS intensities of P(E-A)-*0.1*-*z* mixtures in D_2_O (where *z* = 3:1, 1:1, and 1:3), shown in Figure 4. The SANS intensities of P(E-A)-*0.1*-*z* mixtures in D_2_O (where *z* = 2:1 and 1:2) and P(E-A)-*0.2*-*z* mixtures in D_2_O (where *z* = 3:1, 2:1, 1:1, 1:2 and 1:3) are shown in Figures S8 and S10. All the SANS intensities of P(E-A)-*y*-*z* mixtures displayed a Q^−0^ behavior in the low Q region below a certain temperature, indicating that the mixture contains spherical particles. As the temperature increases, the SANS intensities evolve into a Q^−*a*^ behavior (where *a* increases from 0 to 2, being highly dependent on the temperature *y* and *z*), This suggests that the P(E-A)-*y*-*z* mixture contains either a single type of particle or a combination of 2~3 types, including spherical and cylindrical particles as well as vesicles. Given that the SANS intensity trends of P(E-A)-*y*-*z* mixtures are similar to those of P(E-A)-*x* mixtures, it strongly supports them exhibiting parallel phase behaviors. Notably, the phase transition in P(E-A)-*y*-*z* mixtures likely occurs due to increasing the overall hydrophobicity, which arises from the reduction in f_hydrophilic_ and heat application. The use of a high ratio of P(EO-AGE)(0.75K-2K) block reduces f_hydrophilic_, inducing a phase transition in the P(E-A)-*y*-*z* mixtures even at lower temperatures. Moreover, an increase in temperature enhances this phase transition synergistically in the P(E-A)-*y*-*z* mixtures.

For the detailed structural analysis of P(E-A)-*y*-*z* mixtures, the model fits with various model functions were also conducted, adapting numerical model functions used in SANS analysis of P(E-A)-*x* mixtures exhibiting various Q^−*a*^ behaviors (where *a* was 0, 0.5 < *a* < 2 and 2)) due to the similar patterns and trends of the scattering intensities of the P(E-A)-*x* mixtures. Then, the scattering intensities of all P(E-A)-*0.1-z* mixtures at varying temperatures were successfully reproduced by using three types of form factors (Figure 4 and Figure S9) (specifically, a core–shell spherical form factor for *z* = 3:1, 2:1, 1:1, 1:2 and 1:3 at 25~55 °C, 25~50 °C, 25~45 °C, 25~35 °C and 25~30 °C, respectively, a combination of core–shell spherical and core–shell cylindrical form factors for 3:1, 2:1, 1:1, 1:2 and 1:3 at 55 °C, 55 °C, 45~50 °C, 40 °C and 35 °C, respectively, core–shell cylindrical form factor and a vesicular form factor with a Gaussian distributed shell for 1:1, 1:2 and 1:3 at 40 °C, 45 °C and 55~60 °C, respectively, and a vesicular form factor for 1:2 and 1:3 at 50~60 °C and 45~60 °C, respectively, were used). For the P(E-A)-*0.2-z* mixtures at varying temperatures, the SANS intensities matched well with the proposed form factors, as in the P(E-A)-*0.1-z* mixtures, except for the phase transition temperature, showing that the total concentration of the mixtures similarly influenced their phase transitions (Figure S10). It should be noted that the dispersity of the radius for the vesicle only ranged from 0.31 to 0.59, indicating a partially narrow size distribution (Tables S3 and S4).

**Figure 5:**
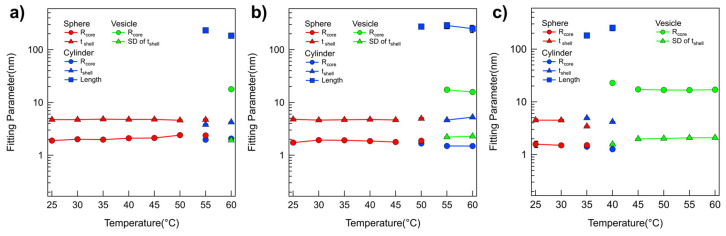
Model fitting results derived from SANS data analysis. Fitting parameters obtained from the form factor analyses of SANS intensities of the P(E-A)-*0.1*-*z* mixtures for (**a**) *z* = 3:1, (**b**) 1:1, and (**c**) 1:3 as the temperature increases from 25 °C to 60 °C. (Error bars are included in every point, but they may not be visible because they are smaller than the symbol.).

The fitting parameters obtained from the SANS analyses of P(E-A)-*y*-*z* mixtures are depicted in Figure 5, Figures S9 and S11. As the P(EO-AGE)(0.75K-2K) blocks adjusted from 3:1 to 1:3, the phase transition temperature, either a spherical to a cylindrical micelle or a cylindrical micelle to a vesicle, generally decreased. This decrease was attributed to the reduction of f_hydrophilic_. The dimension and characteristics (radius, shell thickness, length) of P(E-A)-*y*-*z* mixtures were comparable to those of P(E-A)-*x* mixtures, further supporting that both P(E-A)-*y*-*z* and P(E-A)-*x* mixtures exhibit similar phase behavior. Therefore, this phase behavior also arises from a coupled contribution of P(EO-AGE)(0.75K-2K) (the decrease of f_hydrophilic_) and temperature (the increase in hydrophobicity).

**Figure 6:**
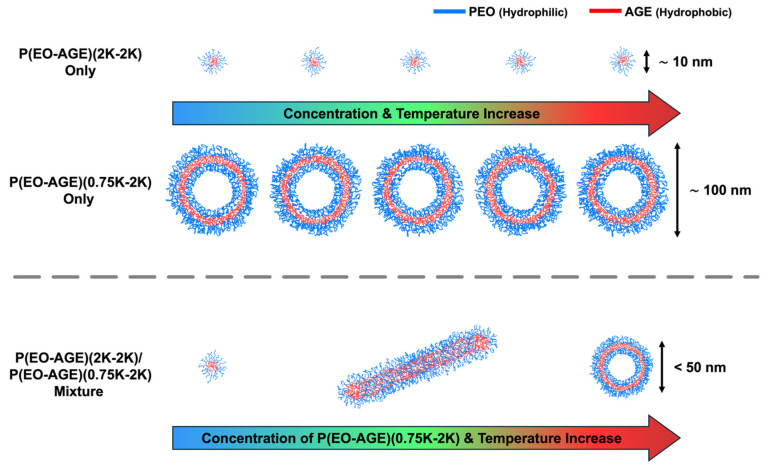
Schematic illustration of the phase transition of the micellar structure of P(EO-AGE)(2K-2K), P(EO-AGE)(0.75K-2K), and P(EO-AGE)(2K-2K)/P(EO-AGE)(0.75K-2K) mixture depending on concentration and temperature.

From the SANS measurements, it was confirmed that the spherical micelles of P(E-A)-*x* and P(E-A)-*y*-*z* mixtures transform into cylindrical micelles, subsequently evolving into unilamellar vesicles depending on the concentration of P(EO-AGE)(0.75K-2K) and temperature. This observation aligns with the findings from DLS measurements. As previously stated, this phase transition is propelled by two factors: an increase in the P(EO-AGE)(0.75K-2K) concentration and temperature, enhancing the effective hydrophobicity of the block copolymer. This causes the effective f_hydrophilic_ of the block copolymer mixture to drop below 0.5. Consequently, the block copolymer mixture transforms from spherical to cylindrical micellar structures and finally to unilamellar vesicles. Notably, at the relatively higher P(EO-AGE)(0.75K-2K) concentration, the temperature sensitivity in the micellar structures increase, facilitating the phase transition at relatively lower temperatures as depicted in Figure 6.

**Figure 7:**
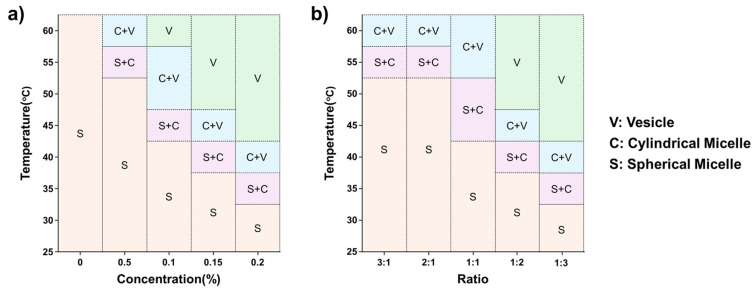
Phase diagram of (**a**) P(E-A)-*x* and (**b**) P(E-A)-*0.1*-*z* mixtures in aqueous solution.

Based on the SANS results for P(E-A)-*x* and P(E-A)-*y*-*z* mixtures, their phase diagrams are visualized as a function of temperature and the relative concentration of P(EO-AGE)(0.75K-2K), as depicted in Figure 7 and Figure S12. The structure of P(E-A)-*x* and P(E-A)-*y*-*z* mixtures is heavily influenced by both temperature and the concentration of P(EO-AGE)(0.75K-2K), forming a unilamellar polymeric vesicle structure at relatively high temperatures and concentrations of P(EO-AGE)(0.75K-2K).

## 4. Conclusions

We explored a straightforward method to fabricate spontaneous unilamellar polymeric vesicles of nanometer scale (<50 nm) without requiring complex preparation procedures. This was achieved by simply mixing the P(EO-AGE)(2K-2K) and P(EO-AGE)(0.75K-2K) in an aqueous solution. We characterized the self-assembling structures of the P(E-A)-*x* and P(E-A)-*y*-*z* mixtures by using dynamic light scattering (DLS) and small-angle neutron scattering (SANS). As the temperature and relative concentration of P(EO-AGE)(0.75K-2K) increased, the phase of the P(E-A)-*x* and P(E-A)-*y*-*z* mixtures transformed from spherical and cylindrical micelles to unilamellar vesicles due to a reduction in the f_hydrophilic_. Consequently, the phase diagrams of the P(E-A)-*x* and P(E-A)-*y*-*z* mixtures were established as a function of temperature and the relative concentration of the P(EO-AGE)(0.75K-2K). Given that the temperature for the micelle-to-vesicle phase transition can be readily adjusted by varying the relative concentration of P(EO-AGE)(0.75K-2K), we anticipate that the fabricated unilamellar polymeric vesicles could serve as thermo-responsive nanocarriers with adjustable temperatures or as a nanoscale reactor for catalysts or enzymes. In particular, it is possible to conduct highly reproducible drug delivery research when applied to drug delivery because it has a unilamellar structure and a narrow size distribution. In addition, since it is a small vesicle of less than 50 nm, it is expected to be able to generate new research results that were not known with a large-sized vesicle (>100 nm). Furthermore, this finding may offer a novel perspective on fabricating the nano-sized building blocks with various structures through a simple process.

## Data Availability

The original contributions presented in this study are included in the article. Further inquiries can be directed to the corresponding author.

## Supplementary Materials

The following supporting information can be downloaded at: https://www.mdpi.com/article/10.3390/mi16101131/s1, Fitting model for SANS analysis; Table S1: Parameters of model fitting of SANS analysis of the P(EO-AGE)(0.75K-2K) with 0.05, 0.1, 0.15 and 0.2 wt% at various temperatures; Table S2: Parameters of model fitting of SANS analysis of P(E-A)-*x* (*x* = 0, 0.05, 0.1, 0.15 and 0.2 wt% in 25~60 °C; Table S3: Parameters of model fitting of SANS analysis of P(E-A)-*0.1*-*z* (z = 1:1, 1:2, 1:3, 2:1 and 3:1 in 25~60 °C); Table S4: Parameters of model fitting of SANS analysis of P(E-A)-*0.2*-*z* (z = 1:1, 1:2, 1:3, 2:1 and 3:1 in 25~60 °C); Figure S1: Scheme for the synthesis of the P(EO-AGE) block copolymers; Figures S2 and S3: ^1^H-NMR spectra and GPC traces of the P(EO-AGE) block copolymers; Figures S4 and S5: SANS intensities and fitting parameters of the P(EO-AGE)(0.75K-2K) with 0.05, 0.1, 0.15 and 0.2 wt% at various temperatures; Figures S6 and S7: SANS intensities and fitting parameters of the P(E-A)-*x* mixtures (*x* = 0.05, 0.15 and 0.2 wt%) at various temperatures; Figures S8 and S9: SANS intensities and fitting parameters of the P(E-A)-*0.1*-*z* mixtures in D_2_O with different ratio (*z* = 2:1 and 1:2) upon heating; Figures S10 and S11: SANS intensities and fitting parameters of P(EO-AGE)(2K-2K) with 0.2 wt% and P(E-A)-*0.2*-*z* mixtures in D_2_O with varying *z* = 3:1, 2:1, 1:1, 1:2 and 1:3 upon heating; Figure S12: Phase diagram of P(E-A)-*0.2*-*z* mixtures in aqueous solution. Reference [53] are cited in the supplementary materials.
