# Supplementary material for: Toward Thermally Stimuli-Responsive Polymeric Vesicles Fabricated by Block Copolymer Blends for Nanocarriers
Source: Micromachines (Basel). 2025 Sep 30;16(10):1131. doi: 10.3390/mi16101131 (PMC12566019; doi:10.3390/mi16101131)
Supplement: Supplementary file 1 [file micromachines-16-01131-s001.zip › micromachines-3872984-supplementary.pdf]

Supporting Information

Toward Thermally Stimuli-Responsive Polymeric Vesicle Fabricated by Block Copolymer Blends for Nanocarriers

Jun-Ki Lee, Seung-Bum Heo, Jong Dae Jang, Dong-Chul Yang, Dae-Hee Yoon, Changwoo Do and Tae-Hwan Kim*

- SANS Analysis

Since the SANS experiments were performed in dilute solutions of particles, the interparticle interactions are negligible. Therefore, the scattering intensity can be simplified as

$$I(q) = nP(q) + b$$

where n is the number of density of particles, $P(q)$ is the intraparticle interference (called the form factor) and b is the residual incoherent scattering.

In this study, we used various model functions to analyze the SANS intensities (core-shell sphere, core-shell cylinder and vesicle with a Gaussian distributed shell). For the SANS analyses, the SANS analysis software (v8.01) provided by NIST center for neutron research was installed and used in Igor Pro (v8.04).

- Core-Shell Sphere

The core-shell sphere form factor is averaged over a Schulz distribution of core radius, where the Schulz distribution can be used to descirbe the distribution of particle sizes in the block copolymer and colloidal systems [1]. The Schulz width parameter is given as $z = 1/p^2 - 1$, where p is the polydispersity ($= \sigma_r/r$). The form factor for the core-shell sphere structures is given as following

$$P(q) = \frac{1}{V_s}\left[\frac{3V_c(\rho_c - \rho_s)j_1(qr_c)}{qr_c} + \frac{3V_s(\rho_s - \rho_{solv})j_1(qr_s)}{qr_s}\right]$$

where r_c and t are the core radius and shell thickness of spherical micelles, respectively. ρ_c, ρ_s and ρ_{solv} are scattering length densities of the core, shell and solvent, respectively.

$$j_1 = (sinx - xconx)/x^2,\ r_s = r_c + t,\ V_s = (\tfrac{4\pi}{3})r_s^3,\ V_c = \left(\tfrac{4\pi}{3}\right) < r_c^3 >,$$
$$\text{and} < r_c^3 > = \tfrac{(z+3)(z+2)}{(z+1)^2} < r_c >.$$

- Core-Shell Cylinder

The core-shell cylindrical form factor is averaged over a Schulz distribution of the length. The Schulz width parameter is given as $z = 1/p^2 - 1$, where p is the polydispersity ($= \sigma_L/L$). The form factor for the core-shell cylindrical structures is given as following

$$P(q)_{cs} = \int_0^{\frac{\pi}{2}} f_{cs}^2(q,\alpha)\, sin\, \alpha\, d\alpha$$

$$f_{cs}(q,\alpha) = 2(\rho_{core} - \rho_{shell})V_{core}j_0\left(\frac{qL_{core}\cos\alpha}{2}\right)\frac{J_1(qR_{core}\sin\alpha)}{(qR_{core}\sin\alpha)} + 2(\rho_{shell} - \rho_{solv})V_{shell}j_0(q(\frac{L_{core}}{2} + t)\cos\alpha)\frac{J_1(q(R_{core}+t)\sin\alpha)}{(q(R_{core}+t)\sin\alpha)}$$

$$j_0 = sin(x)/x$$

$$V = \pi R^2 L, V_{core} = \pi R_{core}^2 L_{core}, V_{shell} = \pi(R_{core} + t)^2(L_{core} + 2t)$$

L and R are the length and radius of the cylindrical particle. L_{core} and R_{core} are the length and radius of the core and t is the shell thickness. ρ_{core}, ρ_{shell} and ρ_{solv} are scattering length densities of the core and shell of core-shell particles and solvent, respectively. $J_1(x)$ is the first order Bessel function and α is the angle between the cylinder axis and the scattering vector, q.

- Vesicle

For the vesicle, we used the form factor with a gaussian distributed shell as following

$$P(q) = 16\pi^2(\Delta\varrho)^2(\delta^2/q^2)\, e^{-q^2t^2}\big(t_1(q) + t_2(q) + t_3(q) + t_4(q)\big)$$

$$t_1(q) = \frac{1}{2}q^2t^4\big(1 + \cos 2qr_0 e^{-2\sigma^2q^2}\big)$$

$$t_2(q) = qt^2(r_0 \sin 2qr_0 + 2q\sigma^2 \cos 2qr_0)e^{-2\sigma^2q^2}$$

$$t_3(q) = \frac{1}{2}r_0^2\big(1 - \cos 2qr_0 e^{-2\sigma^2q^2}\big)$$

$$t_4(q) = \frac{1}{2}\sigma^2\big(1 + 4qr_0 \sin 2qr_0 e^{-2\sigma^2q^2} + \cos 2qr_0(4\sigma^2q^2 - 1)e^{-2\sigma^2q^2}\big)$$

where r_0, t and δ are the core radius, the standard deviation of the Gaussian scattering length density profile and $t\sqrt{2\pi}$, respectively.

- Parameters for Model fitting of SANS analysis

Table S1. P(EO-AGE)(0.75K-2K) with 0.05, 0.1, 0.15 and 0.2 wt% in 25 ~ 60 °C.

**0.05 wt%**	SLD (Å^{-2})	Polydispersity<br>Vesicle
25 °C	$3.17x10^{7}$±$2.16x10^{8}$	0.47 ± 0.03
30 °C	$3.17x10^{7}$±$1.60x10^{8}$	0.55 ± 0.03
35 °C	$3.17x10^{7}$±$1.08x10^{8}$	0.66 ± 0.03
40 °C	$3.15x10^{7}$±$8.52x10^{9}$	0.60 ± $0.01x10^{-1}$
45 °C	$3.15x10^{7}$±$7.78x10^{9}$	0.60 ± $0.01x10^{-1}$
50 °C	$3.17x10^{7}$±$7.41x10^{9}$	0.60 ± $0.01x10^{-1}$
55 °C	$3.15x10^{7}$±$7.04x10^{9}$	0.60 ± $0.01x10^{-1}$
60 °C	$3.17x10^{7}$±$6.93x10^{9}$	0.67 ± 0.02
**0.10 wt%**	SLD (Å^{-2})	Polydispersity<br>Vesicle
25 °C	$3.17x10^{7}$±$9.53x10^{9}$	0.34 ± $0.07x10^{-1}$
30 °C	$3.17x10^{7}$±$8.25x10^{9}$	0.29 ± $0.05x10^{-1}$
35 °C	$3.17x10^{7}$±$6.65x10^{9}$	0.31 ± $0.04x10^{-1}$
40 °C	$3.17x10^{7}$±$5.73x10^{9}$	0.31 ± $0.04x10^{-1}$
45 °C	$3.17x10^{7}$±$5.52x10^{9}$	0.33 ± $0.04x10^{-1}$
50 °C	$3.17x10^{7}$±$5.41x10^{9}$	0.32 ± $0.04x10^{-1}$
55 °C	$3.17x10^{7}$±$5.13x10^{9}$	0.33 ± $0.04x10^{-1}$
60 °C	$3.17x10^{7}$±$4.95x10^{9}$	0.33 ± $0.04x10^{-1}$
**0.15 wt%**	SLD (Å^{-2})	Polydispersity<br>Vesicle
25 °C	$3.17x10^{7}$±$6.91x10^{9}$	0.30 ± $0.04x10^{-1}$
30 °C	$3.17x10^{7}$±$6.28x10^{9}$	0.34 ± $0.05x10^{-1}$
35 °C	$3.17x10^{7}$±$5.32x10^{9}$	0.36 ± $0.05x10^{-1}$
40 °C	$3.17x10^{7}$±$4.79x10^{9}$	0.37 ± $0.05x10^{-1}$
45 °C	$3.17x10^{7}$±$4.64x10^{9}$	0.39 ± $0.05x10^{-1}$
50 °C	$3.17x10^{7}$±$4.51x10^{9}$	0.40 ± $0.05x10^{-1}$
55 °C	$3.17x10^{7}$±$4.37x10^{9}$	0.39 ± $0.05x10^{-1}$
60 °C	$3.17x10^{7}$±$4.29x10^{9}$	0.38 ± $0.05x10^{-1}$
**0.20 wt%**	SLD (Å^{-2})	Polydispersity<br>Vesicle
25 °C	$3.17x10^{7}$±$5.39x10^{9}$	0.30 ± $0.03x10^{-1}$
30 °C	$3.17x10^{7}$±$4.89x10^{9}$	0.33 ± $0.03x10^{-1}$
35 °C	$3.17x10^{7}$±$4.32x10^{9}$	0.35 ± $0.04x10^{-1}$
40 °C	$3.17x10^{7}$±$4.02x10^{9}$	0.36 ± $0.04x10^{-1}$
45 °C	$3.17x10^{7}$±$3.88x10^{9}$	0.38 ± $0.04x10^{-1}$
50 °C	$3.17x10^{7}$±$3.78x10^{9}$	0.38 ± $0.04x10^{-1}$
55 °C	$3.17x10^{7}$±$3.74x10^{9}$	0.38 ± $0.04x10^{-1}$
60 °C	$3.17x10^{7}$±$1.04x10^{8}$	0.38 ± $0.01x10^{-1}$

Table S2. P(E-A)-x (x = 0, 0.05, 0.1, 0.15 and 0.2 wt% in 25 ~ 60 °C)

***x* = 0**	SLD (Å^{-2})	Polydispersity		
		Sphere		
25 °C	$3.08x10^{-6} \pm 6.44x10^{-8}$	0.50 ± 0.01		
30 °C	$3.07x10^{-6} \pm 6.22x10^{-8}$	0.48 ± 0.01		
35 °C	$2.94x10^{-6} \pm 5.91x10^{-8}$	0.44 ± 0.01		
40 °C	$3.01x10^{-6} \pm 5.80x10^{-8}$	0.46 ± 0.01		
45 °C	$2.81x10^{-6} \pm 5.87x10^{-8}$	0.41 ± 0.01		
50 °C	$2.71x10^{-6} \pm 5.67x10^{-8}$	0.40 ± 0.01		
55 °C	$2.56x10^{-6} \pm 5.49x10^{-8}$	0.38 ± 0.01		
60 °C	$2.48x10^{-6} \pm 4.22x10^{-8}$	0.42 ± 0.01		
***x* = 0.05**	SLD (Å^{-2})	Polydispersity (Relative Fraction)		
		Sphere	Cylinder	Vesicle
25 °C	$2.34x10^{-6} \pm 1.23x10^{-7}$	0.61 ± 0.04		
30 °C	$2.33x10^{-6} \pm 1.23x10^{-7}$	0.51 ± 0.03		
35 °C	$2.20x10^{-6} \pm 1.08x10^{-7}$	0.47 ± 0.03		
40 °C	$2.16x10^{-6} \pm 1.06x10^{-7}$	0.45 ± 0.03		
45 °C	$2.18x10^{-6} \pm 1.06x10^{-7}$	0.44 ± 0.03		
50 °C	$2.25x10^{-6} \pm 8.27x10^{-8}$	0.50 ± 0.02		
55 °C	$3.55x10^{-6} \pm 4.39x10^{-9}$	0.57 ± 0.01 (55 %)	0.10 ± 0.02 (45 %)	
60 °C	$4.08x10^{-7} \pm 4.78x10^{-9}$		0.29 ± 0.02 (35 %)	0.28 ± 0.03 x10^{-1} (65 %)
***x* = 0.1**	SLD (Å^{-2})	Polydispersity (Relative Fraction)		
		Sphere	Cylinder	Vesicle
25 °C	$1.46x10^{-6} \pm 1.16x10^{-7}$	0.51 ± 0.04		
30 °C	$1.88x10^{-6} \pm 1.07x10^{-7}$	0.52 ± 0.03		
35 °C	$1.94x10^{-6} \pm 9.94x10^{-8}$	0.51 ± 0.03		
40 °C	$2.01x10^{-6} \pm 8.60x10^{-8}$	0.55 ± 0.03		
45 °C	$2.00x10^{-6} \pm 6.85x10^{-9}$	0.49 ± 0.01 (23 %)	0.11 ± 0.02 (77 %)	
50 °C	$3.16x10^{-7} \pm 2.25x10^{-8}$		0.46 ± 0.03x10^{-1} (78 %)	0.24 ± 0.01 (22 %)
55 °C	$1.88x10^{-7} \pm 3.84x10^{-9}$		0.38 ± 0.02 (22 %)	0.46 ± 0.06x10^{-1} (78 %)
60 °C	$2.88x10^{-7} \pm 3.41x10^{-9}$			0.41 ± 0.01x10^{-1}

***x* = 0.15**	SLD (Å^{-2})	Polydispersity (Relative Fraction)		
		Sphere	Cylinder	Vesicle
25 °C	$1.38 \times 10^{6} \pm 1.03 \times 10^{7}$	0.49 ± 0.04		
30 °C	$1.49 \times 10^{6} \pm 9.12 \times 10^{8}$	0.45 ± 0.03		
35 °C	$1.40 \times 10^{6} \pm 6.43 \times 10^{8}$	0.52 ± 0.02		
40 °C	$1.33 \times 10^{6} \pm 1.06 \times 10^{8}$	0.60 ± 0.02 (17 %)	0.40 ± 0.03x10^{-1} (83 %)	
45 °C	$3.17 \times 10^{7} \pm 3.29 \times 10^{8}$		0.47 ± 0.04x10^{-1} (76 %)	0.31 ± 0.01 (24 %)
50 °C	$2.44 \times 10^{7} \pm 3.34 \times 10^{9}$			0.45 ± 0.06x10^{-2}
55 °C	$2.38 \times 10^{7} \pm 3.24 \times 10^{9}$			0.50 ± 0.05x10^{-2}
60 °C	$2.53 \times 10^{7} \pm 3.14 \times 10^{9}$			0.50 ± 0.05x10^{-2}

***x* = 0.2**	SLD (Å^{-2})	Polydispersity (Relative Fraction)		
		Sphere	Cylinder	Vesicle
25 °C	$1.00 \times 10^{6} \pm 1.09 \times 10^{7}$	0.48 ± 0.04		
30 °C	$1.14 \times 10^{6} \pm 9.92 \times 10^{8}$	0.52 ± 0.04		
35 °C	$1.32 \times 10^{6} \pm 1.60 \times 10^{8}$	0.62 ± 0.05 (23 %)	0.35 ± 0.05x10^{-1} (77 %)	
40 °C	$2.85 \times 10^{7} \pm 4.47 \times 10^{8}$		0.28 ± 0.02 (95%)	0.26 ± 0.01 (5%)
45 °C	$3.23 \times 10^{7} \pm 1.06 \times 10^{8}$			0.26 ± 0.02x10^{-1}
50 °C	$2.48 \times 10^{7} \pm 3.02 \times 10^{9}$			0.43 ± 0.04x10^{-1}
55 °C	$1.94 \times 10^{7} \pm 2.98 \times 10^{9}$			0.45 ± 0.04x10^{-1}
60 °C	$1.65 \times 10^{7} \pm 2.93 \times 10^{9}$			0.45 ± 0.04x10^{-1}

Table S3. P(E-A)-***0.1***-*z* (*z* = 1:1, 1:2, 1:3, 2:1 and 3:1 in 25 ~ 60 °C)

***z* = 3:1**	SLD ($Å^{-2}$)	Polydispersity (Relative Fraction)		
		Sphere	Cylinder	Vesicle
25 °C	$2.72x10^{6}±8.11x10^{8}$	0.52 ± 0.02		
30 °C	$2.75x10^{6}±7.59x10^{8}$	0.49 ± 0.02		
35 °C	$2.67x10^{6}±7.56x10^{8}$	0.49 ± 0.02		
40 °C	$2.57x10^{6}±7.35x10^{8}$	0.45 ± 0.02		
45 °C	$2.55x10^{6}±7.17x10^{8}$	0.45 ± 0.02		
50 °C	$2.40x10^{6}±6.09x10^{8}$	0.41 ± 0.01		
55 °C	$5.31x10^{6}±1.19x10^{9}$	0.29 ± 0.01 (89 %)	0.14 ± 0.04 (11 %)	
60 °C	$3.83x10^{7}±1.36x10^{8}$		0.10 ± 0.01 (82 %)	$0.19 ± 0.04x10^{-1}$ (18 %)
***z* = 2:1**	SLD ($Å^{-2}$)	Polydispersity (Relative Fraction)		
		Sphere	Cylinder	Vesicle
25 °C	$2.63x10^{6}±8.07x10^{8}$	0.50 ± 0.02		
30 °C	$2.59x10^{6}±7.65x10^{8}$	0.48 ± 0.02		
35 °C	$2.55x10^{6}±7.26x10^{8}$	0.48 ± 0.02		
40 °C	$2.62x10^{6}±6.72x10^{8}$	0.47 ± 0.02		
45 °C	$2.47x10^{6}±6.38x10^{8}$	0.43 ± 0.01		
50 °C	$2.37x10^{6}±6.1x10^{8}$	0.43 ± 0.01		
55 °C	$3.58x10^{6}±6.35x10^{9}$	0.37 ± 0.01 (55 %)	0.16 ± 0.03 (45 %)	
60 °C	$4.54x10^{7}±1.81x10^{8}$		$0.10 ± 0.07x10^{1}$ (79 %)	$0.18 ± 0.03x10^{1}$ (21%)
***z* = 1:1**	SLD ($Å^{-2}$)	Polydispersity (Relative Fraction)		
		Sphere	Cylinder	Vesicle
25 °C	$2.27x10^{6}±1.10x10^{7}$	0.55 ± 0.03		
30 °C	$2.20x10^{6}±1.02x10^{7}$	0.49 ± 0.03		
35 °C	$2.32x10^{6}±9.64x10^{8}$	0.49 ± 0.02		
40 °C	$2.34x10^{6}±8.08x10^{8}$	0.55 ± 0.02		
45 °C	$2.45x10^{6}±6.52x10^{8}$	0.71 ± 0.02		
50 °C	$3.67x10^{6}±3.83x10^{7}$	0.88 ± 0.02 (44 %)	0. 41 ± 0.01 (56 %)	
55 °C	$3.51x10^{7}±4.69x10^{9}$		0.10 ± 0.02 (8 %)	$0.61 ± 0.08x10^{2}$ (92 %)
60 °C	$3.14x10^{7}±4.00x10^{9}$		0.10 ± 0.04 (4 %)	$0.59 ± 0.06x10^{2}$ (96 %)

z = 1:2	SLD (Å^{-2})	Polydispersity (Relative Fraction)		
		Sphere	Cylinder	Vesicle
25 °C	$1.44x10^{6}±1.11x10^{7}$	0.54 ± 0.04		
30 °C	$1.59x10^{6}±9.53x10^{8}$	0.53 ± 0.03		
35 °C	$1.89x10^{6}±8.62x10^{8}$	0.30 ± 0.03		
40 °C	$3.40x10^{6}±2.10x10^{7}$	0.61 ± 0.01 (21 %)	0.58 ± 0.01 (79 %)	
45 °C	$3.25x10^{7}±2.89x10^{8}$		0.15 ± 0.01 (61 %)	0.52 ± 0.03 (39 %)
50 °C	$2.36x10^{7}±3.32x10^{9}$			0.59 ± 0.01
55 °C	$2.33x10^{7}±3.22x10^{9}$			0.49 ± 0.53x10^{-3}
60 °C	$2.16x10^{7}±3.15x10^{9}$			0.50 ± 0.52x10^{-3}
***z* = 1:3**	SLD (Å^{-2})	Polydispersity (Relative Fraction)		
		Sphere	Cylinder	Vesicle
25 °C	$1.30x10^{7}±4.62x10^{7}$	0.57 ± 0.07		
30 °C	$1.37x10^{6}±1.09x10^{8}$	0.78 ± 0.05		
35 °C	$1.37x10^{6}±7.88x10^{9}$	0.40 ± 0.02 x10^{-1} (48 %)	0.63 ± 0.01 (52 %)	
40 °C	$6.21x10^{7}±1.56x10^{8}$		0.13 ± 0.02 (19 %)	0.24 ± 0.03x10^{-1} (81 %)
45 °C	$3.07x10^{7}±4.27x10^{9}$			0.46 ± 0.06x10^{-1}
50 °C	$3.07x10^{7}±4.10x10^{9}$			0.46 ± 0.06x10^{-1}
55 °C	$3.07x10^{7}±3.95x10^{9}$			0.49 ± 0.06x10^{-1}
60 °C	$3.07x10^{7}±3.84x10^{9}$			0.48 ± 0.06x10^{-1}

Table S4. P(E-A)-***0.2***-*z* (*z* = 1:1, 1:2, 1:3, 2:1 and 3:1 in 25 ~ 60 °C)

***z* = 3:1**	SLD (Å^{-2})	Polydispersity (Relative Fraction)		
		Sphere	Cylinder	Vesicle
25 °C	$2.94x10^{6}\pm3.83x10^{8}$	0.49 ± 0.01		
30 °C	$2.98x10^{6}\pm3.48x10^{8}$	0.47 ± 0.01		
35 °C	$2.92x10^{6}\pm3.54x10^{8}$	0.47 ± 0.01		
40 °C	$2.78x10^{6}\pm3.60x10^{8}$	0.43 ± 0.01		
45 °C	$2.58x10^{6}\pm2.97x10^{8}$	0.38 ± 0.01		
50 °C	$2.45x10^{6}\pm2.83x10^{8}$	0.36 ± 0.01		
55 °C	$2.75x10^{6}\pm1.67x10^{8}$	$0.40\pm0.02x10^{-1}$ (29 %)	$0.41\pm0.04x10^{-1}$ (71 %)	
60 °C	$4.31x10^{7}\pm1.09x10^{8}$		$0.65\pm0.02x10^{-1}$ (65 %)	$0.60\pm0.02x10^{-1}$ (35 %)
***z* = 2:1**	SLD (Å^{-2})	Polydispersity (Relative Fraction)		
		Sphere	Cylinder	Vesicle
25 °C	$2.71x10^{6}\pm3.91x10^{8}$	0.49784 ± 0.00936		
30 °C	$2.61x10^{6}\pm3.63x10^{8}$	0.45218 ± 0.00834		
35 °C	$2.61x10^{6}\pm3.64x10^{8}$	0.45742 ± 0.00844		
40 °C	$2.66x10^{6}\pm3.71x10^{8}$	0.46 ± 0.00851		
45 °C	$2.55x10^{6}\pm2.73x10^{8}$	0.47531 ± 0.00683		
50 °C	$2.61x10^{6}\pm1.78x10^{8}$	0.58 ± 0.01 (33 %)	0.10 ± 0.01 (67 %)	
55 °C	$4.31x10^{7}\pm2.14x10^{8}$		$0.11\pm0.05x10^{-1}$ (44 %)	$0.56\pm0.02x10^{-1}$ (56 %)
60 °C	$3.23x10^{7}\pm3.11x10^{9}$		0.11 ± 0.03 (8 %)	$0.67\pm0.03x10^{2}$ (92 %)
***z* = 1:1**	SLD (Å^{-2})	Polydispersity (Relative Fraction)		
		Sphere	Cylinder	Vesicle
25 °C	$3.03x10^{6}\pm2.39x10^{8}$	0.64 ± 0.01		
30 °C	$3.09x10^{6}\pm1.99x10^{8}$	$0.60 \pm 0.04x10^{-1}$		
35 °C	$3.02x10^{6}\pm1.92x10^{8}$	$0.59 \pm 0.04x10^{-1}$		
40 °C	$2.84x10^{6}\pm1.92x10^{8}$	$0.66 \pm 0.03x10^{-1}$		
45 °C	$2.79x10^{6}\pm1.61x10^{8}$	0.79 ± 0.03 (44 %)	$0.59 \pm 0.02x10^{-1}$ (56 %)	
50 °C	$2.83x10^{7}\pm3.04x10^{8}$		$0.32 \pm 0.02x10^{-1}$ (92%)	$0.51 \pm 0.05x10^{-1}$ (8 %)
55 °C	$2.09x10^{7}\pm7.38x10^{9}$		0.10 ± 0.04 (7 %)	$0.61 \pm 0.01x10^{-1}$ (93 %)
60 °C	$2.45x10^{7}\pm9.49x10^{9}$		0.57 ± 0.18 (1 %)	$0.61 \pm 0.01x10^{-1}$ (99 %)

z = 1:2	SLD (Å^{-2})	Polydispersity (Relative Fraction)		
		Sphere	Cylinder	Vesicle
25 °C	$1.92x10^{6} \pm 6.73x10^{8}$	0.51 ± 0.02		
30 °C	$2.12x10^{6} \pm 6.1x10^{8}$	0.51 ± 0.02		
35 °C	$2.04x10^{6} \pm 5.94x10^{8}$	0.48 ± 0.02		
40 °C	$2.14x10^{6} \pm 6.04x10^{8}$	0.52 ± 0.02		
45 °C	$3.28x10^{7} \pm 1.20x10^{8}$		0.50 ± 0.03 x10^{-1} (37 %)	0.41 ± 0.07x10^{-1} (63 %)
50 °C	$3.17x10^{7} \pm 2.39x10^{9}$			0.42 ± 0.01x10^{-1}
55 °C	$3.05x10^{7} \pm 2.34x10^{9}$			0.43 ± 0.01x10^{-1}
60 °C	$3.28x10^{7} \pm 2.30x10^{9}$			0.44 ± 0.01x10^{-1}
***z* = 1:3**	SLD (Å^{-2})	Polydispersity (Relative Fraction)		
		Sphere	Cylinder	Vesicle
25 °C	$2.30x10^{6} \pm 1.14x10^{7}$	0.93 ± 0.02		
30 °C	$3.80x10^{6} \pm 7.41x10^{9}$	0.84 ± 0.01 (94 %)	0.50 ± 0.01 (6 %)	
35 °C	$3.80x10^{6} \pm 8.26x10^{7}$	0.76 ± 0.01 (94 %)	0.35 ± 0.01 (6 %)	
40 °C	$3.80x10^{6} \pm 8.31x10^{8}$	0.91 ± 0.03 (95 %)	0.35 ± 0.01 (5 %)	
45 °C	$4.04x10^{7} \pm 2.51x10^{9}$			0.31 ± 0.02x10^{-1}
50 °C	$3.19x10^{7} \pm 2.47x10^{9}$			0.31 ± 0.02x10^{-1}
55 °C	$2.42x10^{7} \pm 2.44x10^{9}$			0.31 ± 0.01x10^{-1}
60 °C	$2.19x10^{7} \pm 2.40x10^{9}$			0.31 ± 0.01x10^{-1}

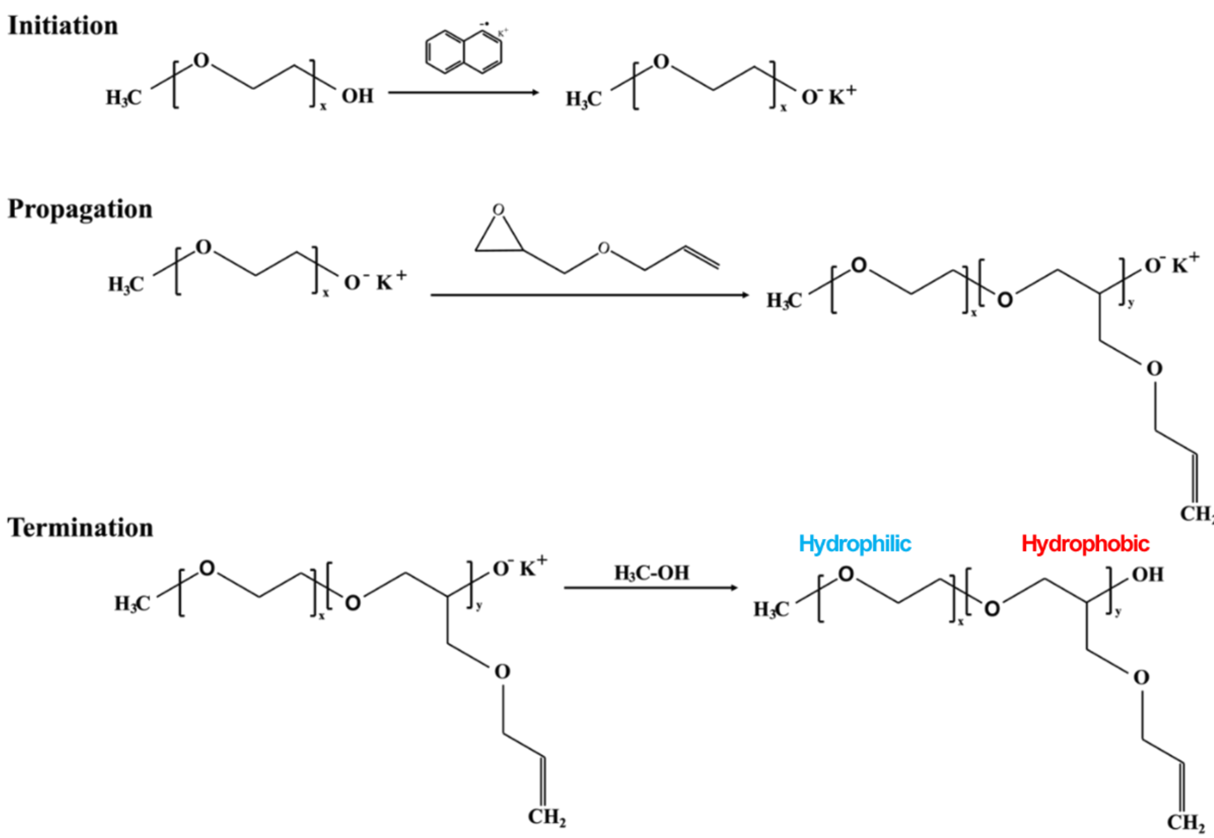

Figure S1. Synthesis scheme of the P(EO-AGE) block copolymers (where x = 46 or 16 and y = 18).

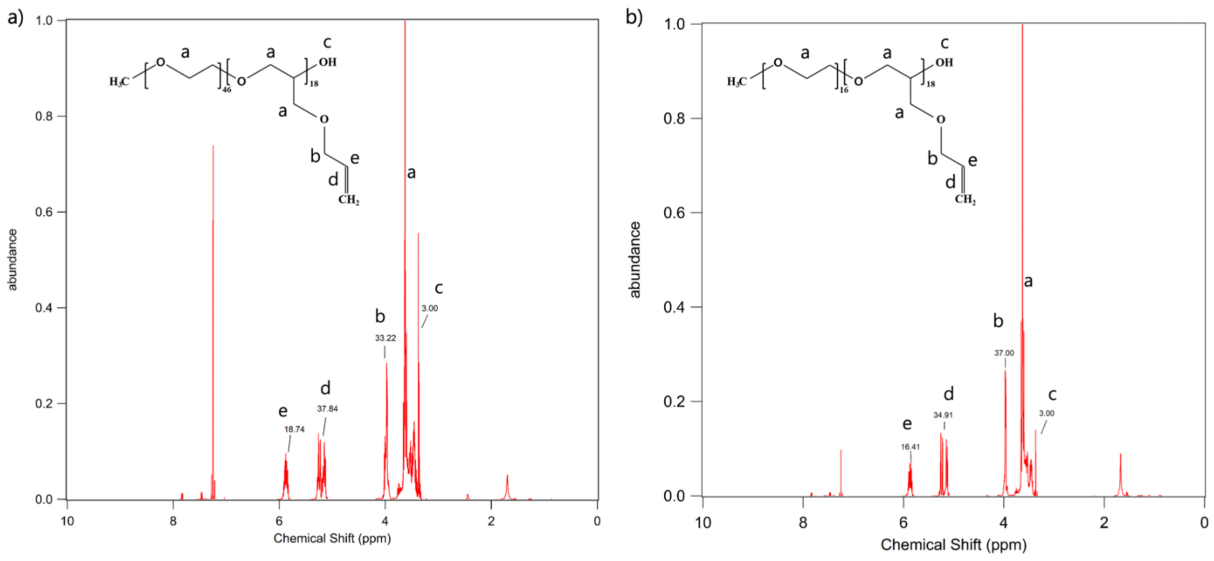

Figure S2. ^{1}H-NMR spectra of the P(EO-AGE) diblock copolymers. ^{1}H-NMR spectra of (a) P(EO-AGE)(2K-2K) and (b) P(EO-AGE)(0.75K-2K) diblock copolymers.

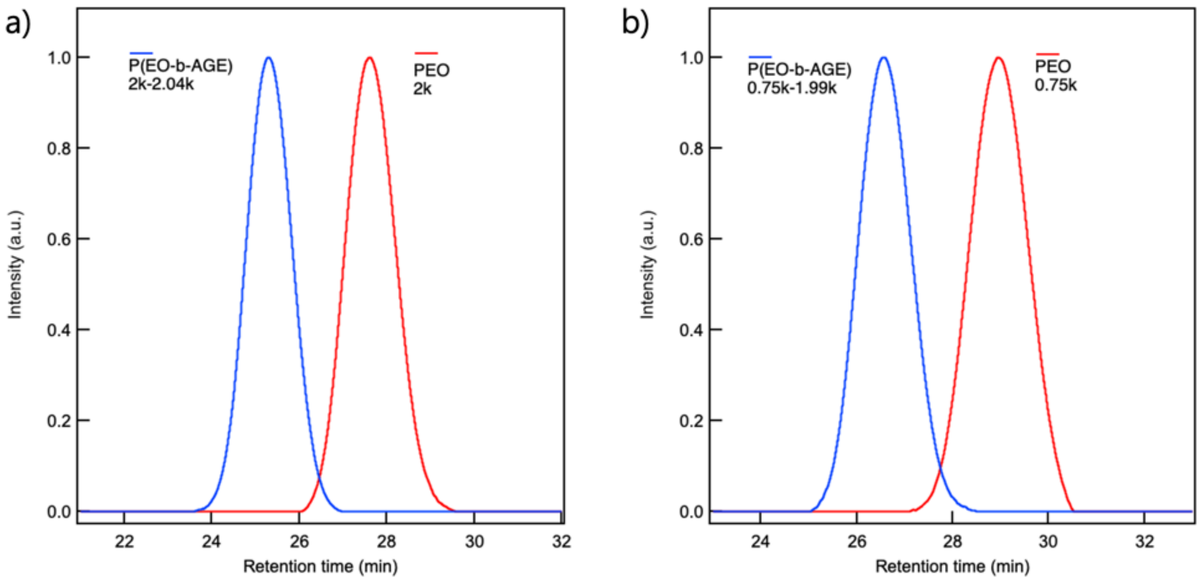

Figure S3. GPC traces of (a) P(EO-AGE)(2K-2K) and (b) P(EO-AGE)(0.75K-2K) diblock copolymers.

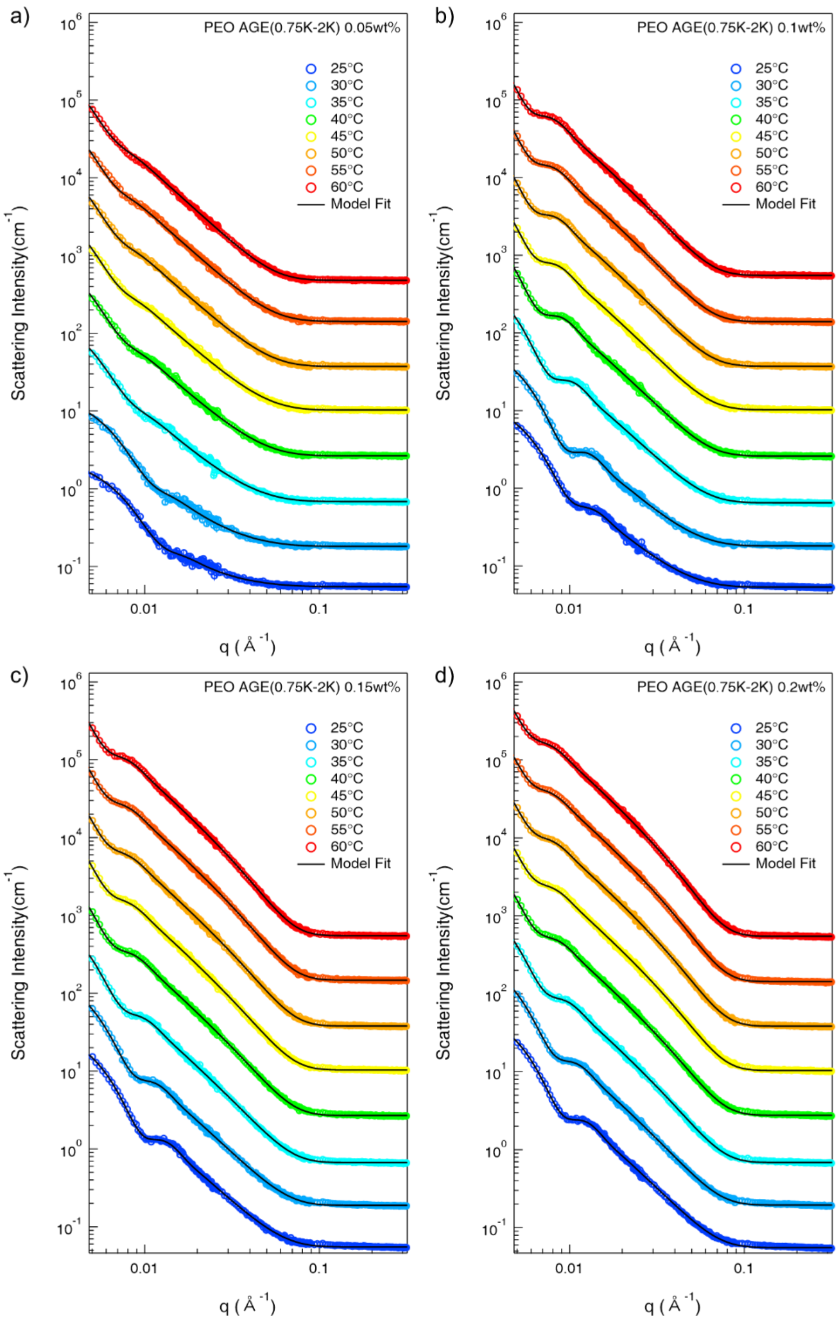

Figure S4. SANS intensities of the P(EO-AGE)(0.75K-2K) with (a) 0.05, (b) 0.1, (c) 0.15 and (d) 0.2 wt% in D_2O during heating (25 ~ 60 °C). SANS intensities have been vertically shifted for better visual clarity.

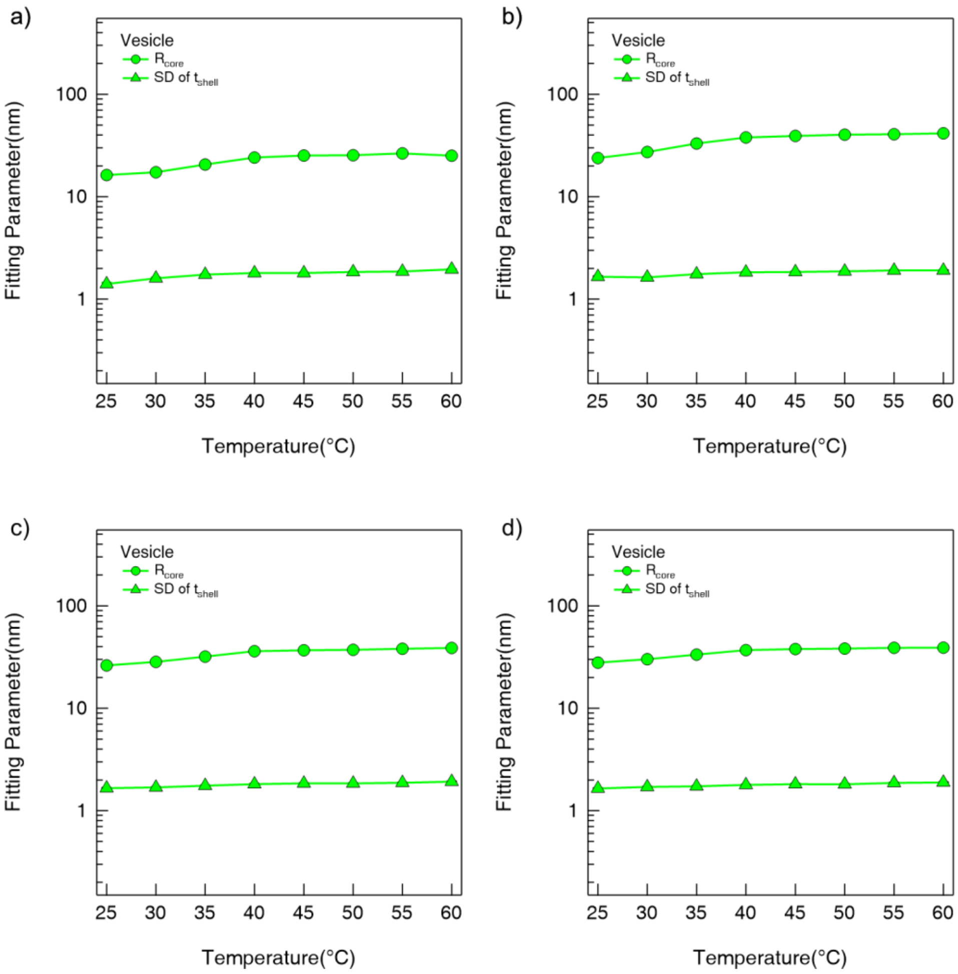

Figure S5. Model fitting results from SANS data analysis. Fitting parameters from the form factor analyses of SANS intensities of P(EO-AGE)(0.75K-2K) with (a) 0.05, (b) 0.1, (c) 0.15 and (d) 0.2 wt% as temperature increases from 25 to 60 °C. (Error bars are included in every point, but they may not be visible because they are smaller than the symbol.)

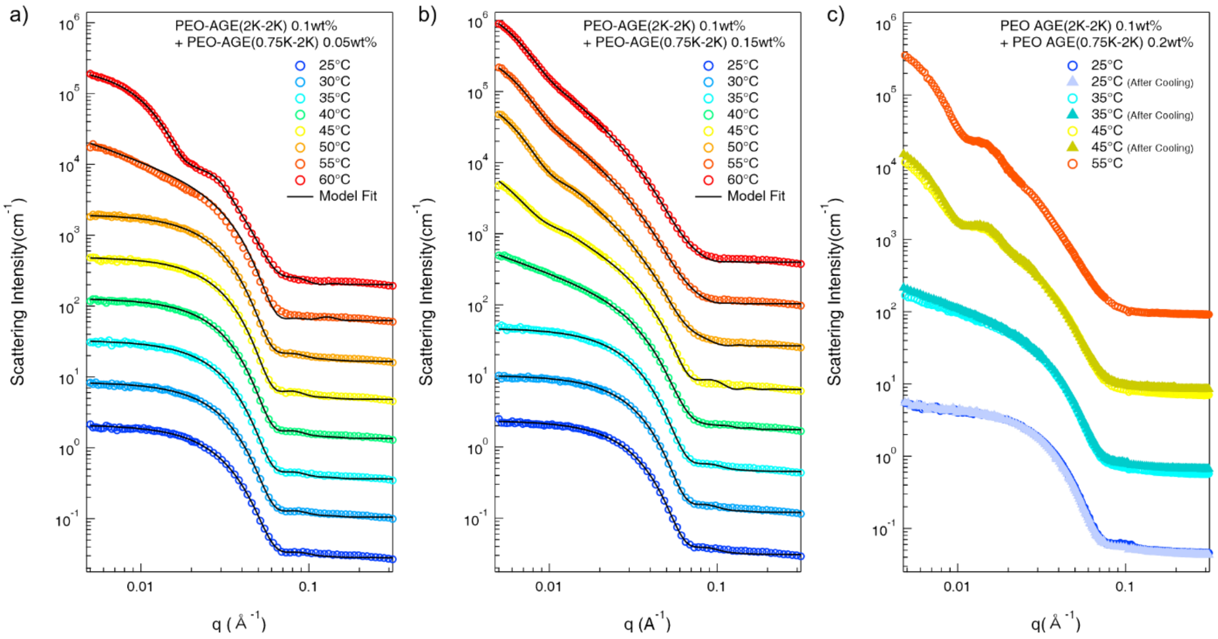

Figure S6. SANS intensities of the P(E-A)-x mixtures at various temperatures (25 to 60 °C) with (a) x = 0.05, (b) x = 0.15 and (c) x = 0.2 wt%. For visual clarity, SANS intensities were vertically shifted.

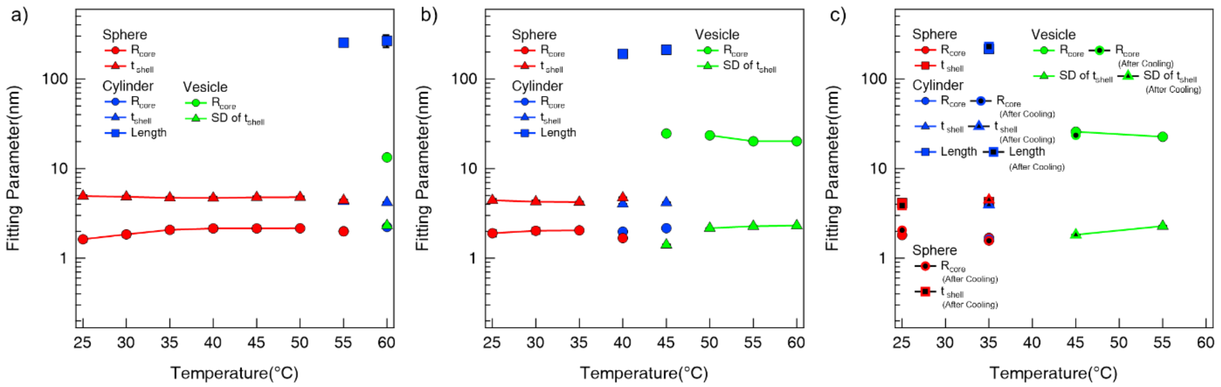

Figure S7. Model fitting results from SANS analysis. Fitting parameters from the form factor fits of SANS intensities of the P(E-A)-x mixtures with (a) x = 0.05, (b) 0.15 and (c) 0.2 wt% when the temperature increases from 25 to 60 °C. (Error bars are included in every point, but they may not be visible because they are smaller than the symbol.)

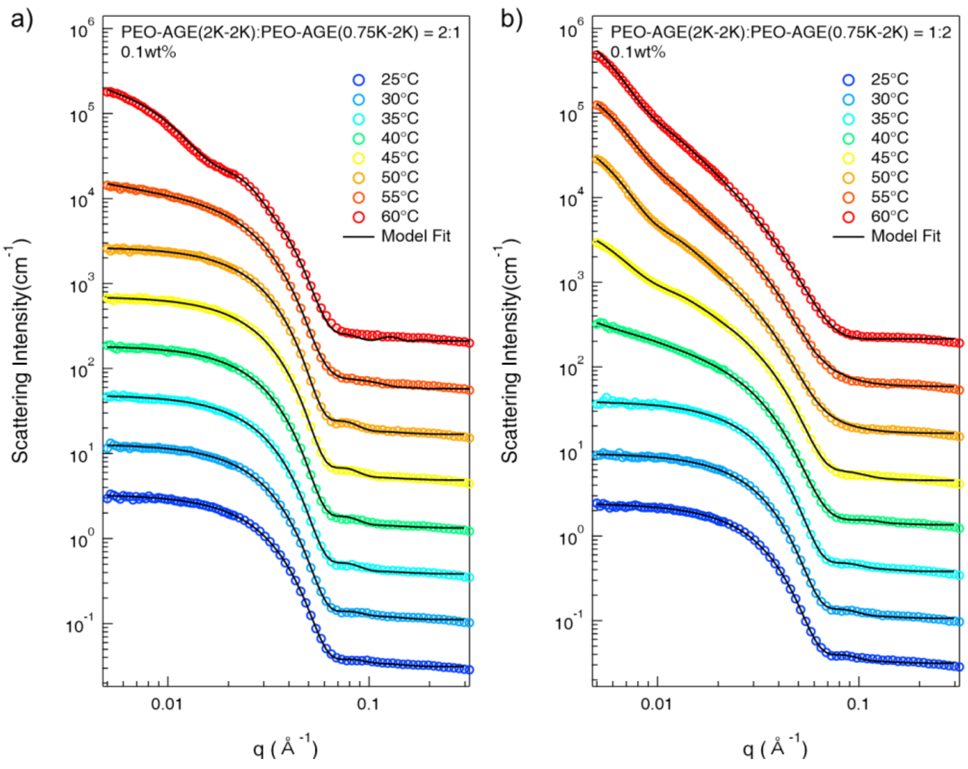

Figure S8. SANS intensities of the P(E-A)-*0.1*-*z* mixtures in D_2O with different (a) *z* = 2:1 and (b) 1:2 upon heating (25 ~ 60 °C). For visual clarity, SANS intensities were vertically shifted.

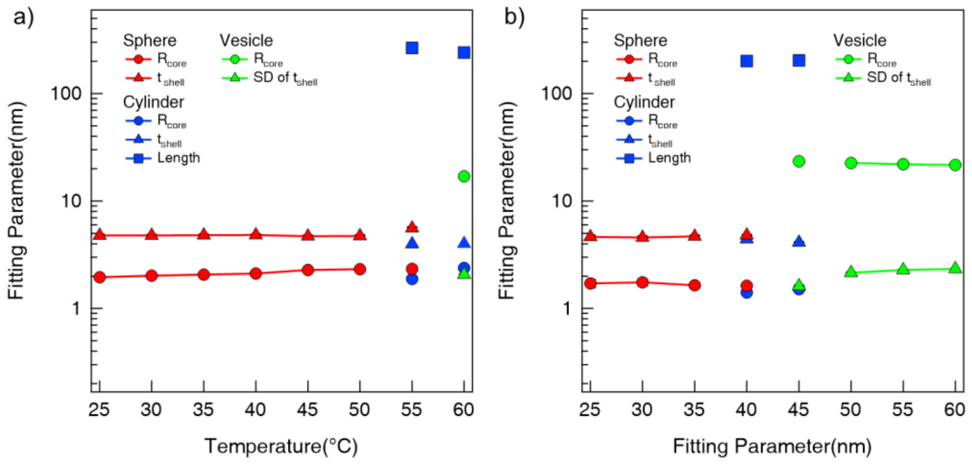

Figure S9. Model fitting results from SANS data analysis. Fitting parameters from the form factor analyses of SANS intensities of the P(E-A)-*0.1*-*z* mixtures with (a) *z* = 2:1 and (b) 1:2 when the temperature increases from 25 to 60 °C. (Error bars are included in every point, but they may not be visible because they are smaller than the symbol.)

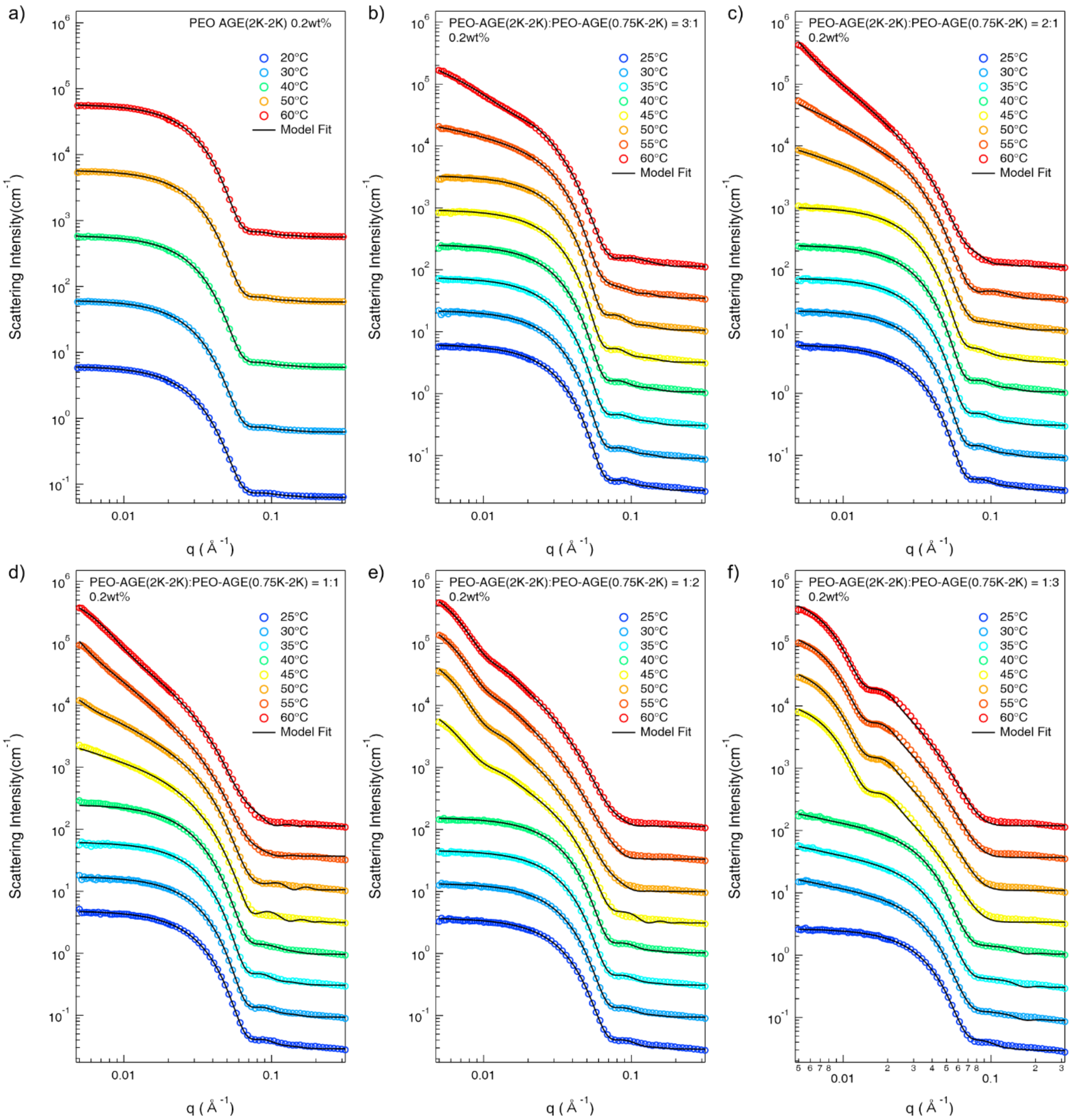

Figure S10. SANS intensities of (a) P(EO-AGE)(2K-2K) with 0.2 wt% and P(E-A)-*0.2*-*z* mixtures in D_2O across various *z* ratios (b) 3:1, (c) 2:1, (d) 1:1, (e) 1:2 and (f) 1:3 during heating (25 ~ 60 °C). SANS intensities have been vertically shifted for better visual clarity.

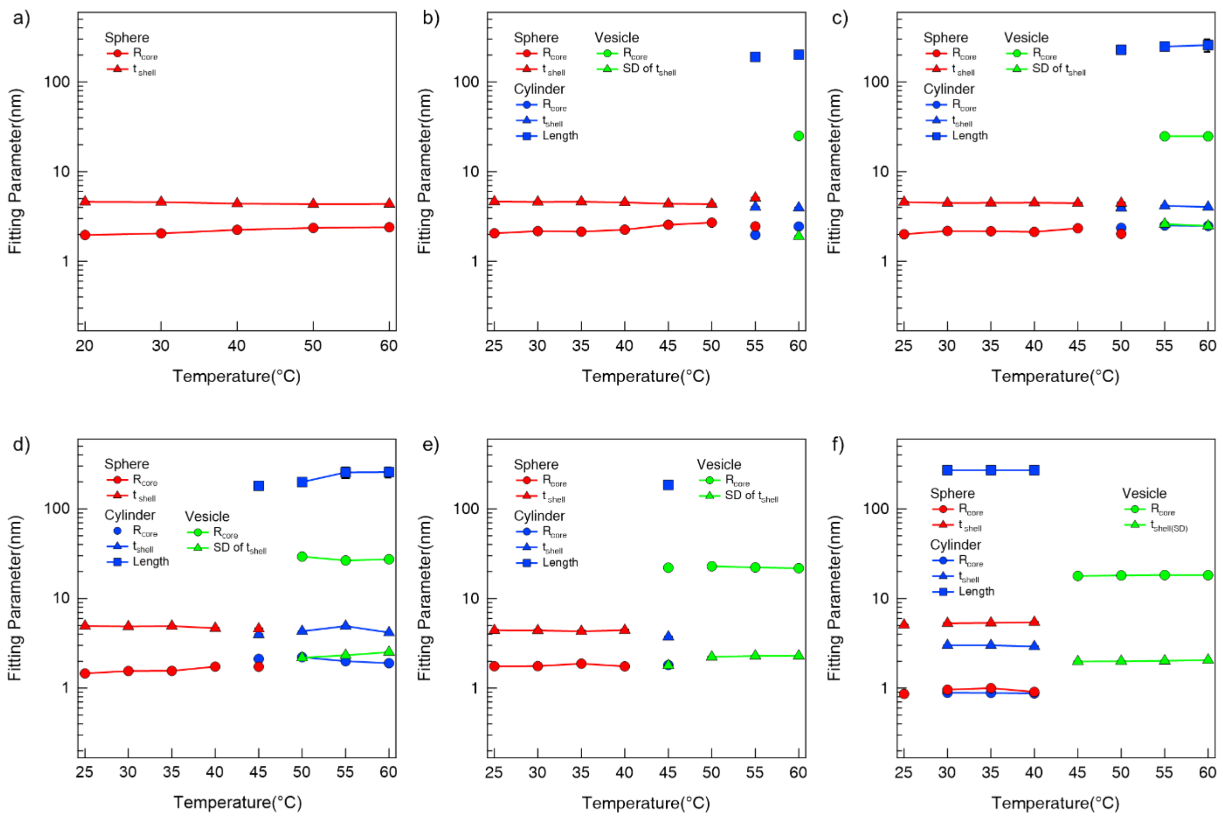

Figure S11. Model fitting results from SANS data analysis. Fitting parameters from the form factor analyses of SANS intensities of (a) P(EO-AGE)(2K-2K) with 0.2 wt% and P(E-A)-*0.2-z* mixtures across various *z* ratios (b) 3:1, (c) 2:1, (d) 1:1, (e) 1:2 and (f) 1:3 as temperature increases from 25 to 60 °C. (Error bars are included in every point, but they may not be visible because they are smaller than the symbol.)

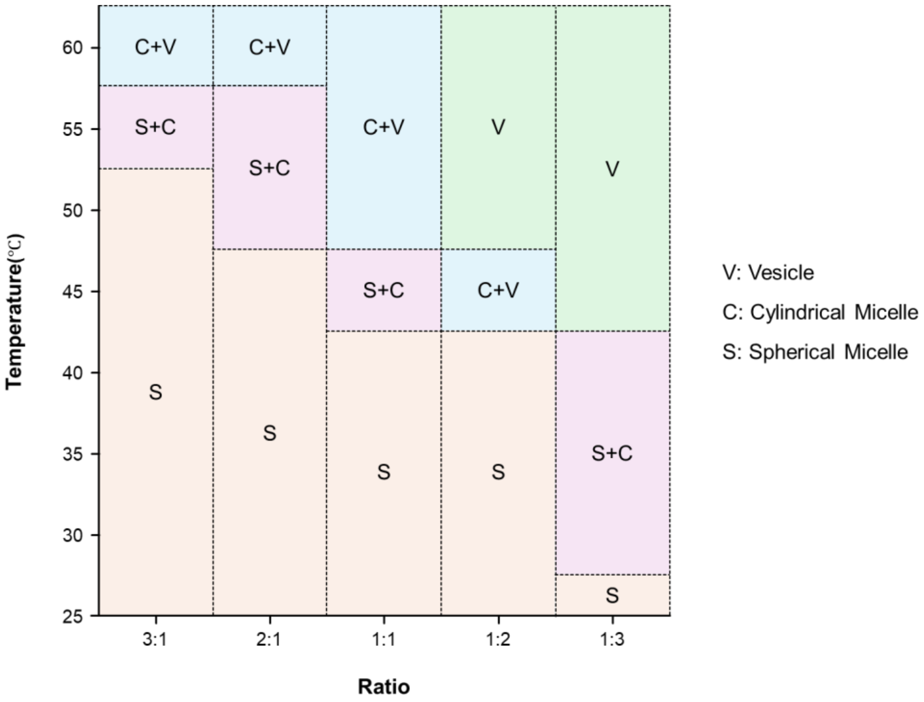

Figure S12. Phase diagram of P(E-A)-*0.2-z* mixtures in aqueous solution.

Reference

1. Schulz, G. V. Z. *Phys. Chem.* **1935**, *43*, 25.
